# Long noncoding RNA SRY-box transcription factor 2 overlapping transcript participates in Parkinson’s disease by regulating the microRNA-942-5p/nuclear apoptosis-inducing factor 1 axis

**DOI:** 10.1080/21655979.2021.1987126

**Published:** 2021-10-20

**Authors:** Yabi Guo, Yanyang Liu, Hong Wang, Peijun Liu

**Affiliations:** Rehabilitation Medicine Center, Xiangyang Central Hospital, Affiliated Hospital of Hubei University of Arts and Science, Xiangyang, China

**Keywords:** LncRNA SOX2-OT, miRNA-942-5p, NAIF1, parkinson’s disease, MPP^+^, SH-SY5Y cells

## Abstract

Parkinson’s disease (PD) is a neurodegenerative disorder. Studies have shown that long noncoding RNA SRY-box transcription factor 2 overlapping transcript (lncRNA SOX2-OT) is highly expressed in PD patients, but its specific functions and mechanisms require further research. To address this gap, this study utilized an *in vitro* PD cell model induced by 1-methyl-4-phenylpyridinium (MPP^+^). Cell viability, apoptosis, lactate dehydrogenase (LDH) activity, inflammatory factor secretion, and oxidative stress indicators were determined by 3-(4,5-dimethylthiazol-2-yl)-2,5-dipheyltetrazolium bromide assay, LDH assay, flow cytometry, enzyme linked immunosorbent assay (ELISA), and corresponding kits, respectively. Gene and protein expression were measured using quantitative real-time-PCR and western blotting, respectively. The results indicated that microRNA-942-5p (miR-942-5p) was a direct target of lncRNA SOX2-OT and nuclear apoptosis-inducing factor 1 (NAIF1) was a direct target of miR-942-5p. The expression levels of lncRNA SOX2-OT and NAIF1 were increased, and miR-942-5p expression was decreased in SH-SY5Y cells following MPP^+^ treatment. In addition, MPP^+^ treatment reduced SH-SY5Y cell viability, increased apoptosis; increased cleaved caspase-3 protein expression and cleaved caspase-3/caspase-3 ratio; enhanced lactate dehydrogenase viability; increased tumor necrosis factor (TNF)-α, interleukin (IL)-1β, and reactive oxygen species, and decreased superoxide dismutase activity in SH-SY5Y cells were inhibited by SOX2-OT-siRNA, and these inhibitions were reversed by miR-942-5p inhibitor. Moreover, the protective role of miR-942-5p mimic in MPP^+^-induced SH-SY5Y cells was eliminated by the NAIF1 plasmid. Overall, lncRNA SOX2-OT-mediated regulation of oxidative stress, inflammation, and neuronal apoptosis were directly controlled by the miR-942-5p/NAIF1 signal axis in MPP^+^-induced SH-SY5Y cells.

## Introduction

Parkinson’s disease (PD) is a universal neurodegenerative disease that results primarily from the death of dopaminergic neurons in the substantia nigra [1,2]. There are many theories regarding the pathogenesis of PD. Patel et al. [3] pointed out that this disease was caused by a combination of age, genetic, and environmental factors indicating that the pathogenesis was complicated. At present, increasing evidence indicates that deficits in mitochondrial function, oxidative stress, inflammation, and apoptosis are the most important factors in PD [4,5], but the specific mechanism is not yet clear. Therefore, it is crucial to study the mechanism of and develop treatment methods for PD.

Long non-coding RNA (lncRNA) play a significant role in the development and diseases of the central nervous system, with a number of lncRNAs being highly expressed in the adult and developing brain [6]. Recent studies have demonstrated that lncRNAs interfere in transcriptional and translational processes, and five lncRNAs are differentially expressed in PD [7]. Moreover, increasing evidence suggests that lncRNA plays a crucial role in the development of PD [8,9]. Long et al. [9] showed that miRNA and lncRNA coregulate the pathogenesis of PD. Lu et al. [10] found that lncRNA MALAT1 targeting miR-4 contributes to cell apoptosis in PD, and the results of a study by Xie et al. [11] showed that upregulated lncRNA small nucleolar RNA host gene 1 promotes 1-methyl-4-phenylpyridinium (MPP^+^)-induced reactive oxygen generation and cytotoxicity in human dopaminergic SH-SY5Y cells through the miR-15b-5p/GSK3β axis. Moreover, SOX2-OT as a lncRNA and some microRNAs (miRNAs) have a targeting relationship in disease [12]. However, the role and specific mechanism of SOX2-OT in PD remain to be investigated.

miRNAs are regarded as types of small non-coding RNAs that modulate gene expression at the post-transcriptional level [13]. In addition, miRNAs can participate in a variety of pathological processes and pathways [14]. Notably, miR-942-5p, as an miRNA, is related to the pathogenesis of many neurodegenerative diseases, such as attention deficit hyperactivity disorder [15]. However, there have been few reports on the role of miR-942-5p in PD. Past studies have shown that cell apoptosis is involved in the pathological mechanisms of PD [16,17]; and the nuclear apoptosis-inducing factor 1 (NAIF1) is crucial in the process of cell apoptosis [18,19]. Moreover, another study has shown that the expression levels of miRNA and NAIF1 are related [20].

Therefore, we hypothesized that lncRNA SOX2-OT might play a facilitating role in PD via regulating miR-942-5p. In this study, we were aimed at exploring whether lncRNA SOX2-OT participates in PD through regulating oxidative stress, inflammation, and neuronal apoptosis by directly regulating miR-942-5p. This study will provide new strategies for PD diagnosis and treatment.

## Materials and methods

### Acquisition and culture of human neuroblastoma SH-SY5Y cells

Human neuroblastoma SH-SY5Y cells were purchased from the American Type Culture Collection (ATCC, Manassas, VA, USA). The cells were stored in Dulbecco’s modified Eagle’s medium (Gibco, Grand Island, NY, USA) supplemented with 1% penicillin/streptomycin and 10% fetal bovine serum (Gibco) and incubated in a humidified atmosphere containing 5% CO_2_ at 37°C.

### *Dual-luciferase reporter verification* [21]

The association between lncRNA SOX2-OT and miR-942-5p was identified by bioinformatics software (Starbase). In addition, TargetScan determined the relationship between miR-942-5p and NAIF1. Then, a dual luciferase reporter gene plasmid vector (Guangzhou RiboBio Co., Ltd., Guangzhou, China) and the QuikChange Site-Directed Mutagenesis Kit (Stratagene, San Diego, CA) were used to generate NAIF1-WT, NAIF1-MUT, SOX2-OT-WT, and SOX2-OT-MUT, according to the manufacturer’s instructions. Finally, the luciferase activity was analyzed by the dual luciferase reporter gene analysis system (Promega, USA).

### Establishment of the PD cell model in vitro

To explore the expression levels of miR-942-5p, lncRNA SOX2-OT, and NAIF1 in the PD cell model, SH-SY5Y cells were treated with 0, 0.25, 0.5, 1, or 2 mM MPP^+^ (Sigma, St. Louis, MO, USA) for 24 h or exposed to 1 mM MPP^+^ for 0, 6, 12, 24, or 48 h [22].

### Cell transfection assay

SH-SY5Y cells were inoculated at a concentration of 5 × 10^4^ cells/mL in six-well plates and incubated overnight. The miR-942-5p inhibitors were used to downregulate miR-942-5p expression in SH-SY5Y cells using an inhibitor control as the negative control. SOX2-OT siRNA was used for SOX2-OT downregulation. The miR-942-5p mimic and NAIF1 plasmid were used to upregulate miR-942-5p and NAIF1 expression in SH-SY5Y cells. Control siRNA, SOX2-OT siRNA, inhibitor control, miR-942-5p inhibitor, SOX2-OT-siRNA + inhibitor control or SOX2-OT-siRNA + miR-942-5p inhibitor, and mimic control, miR-942-5p mimic, control plasmid, NAIF1 plasmid, miR-942-5p mimic + control plasmid or miR-942-5p mimic + NAIF1 plasmid were transfected into SH-SY5Y cells using Lipofectamine 2000 reagent (Invitrogen, USA) according to the manufacturer’s instructions. After 48 h of cell culture at 37°C, the cells were collected to test the transfection efficiency using quantitative real-time (qRT)-PCR, or further cultured in the presence of 1 mM MPP^+^ for 24 h.

### RNA extraction and quantitative real-time PCR

Total cellular RNA was isolated from SH-SY5Y cells using TRIzol reagent (Invitrogen, USA) and reverse-transcribed into first-strand cDNA using a cDNA Synthesis Kit (Invitrogen) according to the instructions provided by the manufacturer. The expression levels of miR-942-5p, lncRNA SOX2-OT, and NAIF1 mRNA were quantified by the Prism 7000 real-time PCR system using Power SYBR Green Master mix (Vazyme, Piscataway, NJ, USA) according to the instructions provided by the manufacturer. The amplification conditions were as follows: denaturation at 94°C for 35 cycles of 60 s, annealing at 60°C for 60 s, and extension at 72°C for 1 min and then at 72°C for 10 min. U6 and GAPDH were used as inner control genes. Calculation of the relative expression levels of miR-942-5p, lncRNA SOX2-OT, and NAIF1 mRNA was undertaken by the 2**^−^**^ΔΔCt^ method [23].

### *3-(4,5-dimethylthiazol-2-yl)-2,5-dipheyltetrazolium bromide (MTT) assay* [24]

SH-SY5Y cells were inoculated into 96-well plates in triplicate and incubated overnight. Subsequently, the medium was removed, and after transfection of the cells at 37°C, the cells were treated with 1 mM MPP^+^. The cells were incubated with 10 μL of MTT solution (Beyotime, Shanghai, China) for 4 h. Subsequently, after removing the solution, 100 μL of dimethyl sulfoxide was added to each well to dissolve the formazan product. Detection was achieved by monitoring the absorbance at 570 nm with a microplate reader (Bio-Rad, Hercules, CA, USA). The optical density value was used to normalize the relative cell viability relative to the control group.

### *Flow cytometry (FCM) to detect apoptosis* [25]

SH-SY5Y cells were seeded into six-well plates overnight and collected by trypsinization following treatment. The cells were washed once with PBS buffer and subsequently re-suspended in 1 × binding buffer. A total of 100 µL of cell suspension was transferred into a 5 mL test tube and mixed with 5 μL of fluorescein isothiocyanate-Annexin V and 5 μL of propidium iodide (BD Biosciences, San Diego, CA), according to the manufacturer’s specifications. The induction of apoptosis was analyzed with a FACSCalibur flow cytometer (BD Biosciences, USA) within 1 h, and data were analyzed with FlowJo software (version 7.6.1; FlowJo LLC).

### *Western blot analysis* [26]

After 48 h of transfection and cell culture, SH-SY5Y cells were treated with 1 mM MPP^+^ for 24 h, washed three times with cold PBS, and immediately lysed with RIPA lysis buffer (Beyotime, Shanghai, China). The lysate was centrifuged at 10,000 x g at 4°C for 10 min, and the total protein level was measured by the BCA protein kit (Pierce, USA). Equal amounts of protein samples were separated using a sodium dodecyl sulfate-polyacrylamide gel electrophoresis gel, and then transferred to PVDF membranes. After sealing with 5% skim milk for 1 h, the PVDF membranes were immediately incubated with NAIF1 (1:1,000; Cell Signaling Technology, Inc., Danvers, MA, USA), cleaved caspase-3 (1:1,000; Cell Signaling Technology, Inc.), caspase 3 (1:1,000; Cell Signaling Technology, Inc.), and GAPDH antibodies (1:1,000; Cell Signaling Technology, Inc.) overnight at 4°C. The following day, the membranes were washed three times with PBST buffer and incubated with horseradish peroxidase-conjugated goat anti-rabbit immunoglobulin G secondary antibody (1:2,000; Cell Signaling Technology, Inc.) at 37°C for 1 h. Finally, the protein bands were visualized using ECL luminescent substrate (Pierce) according to the manufacturer’s instructions. The experiments were repeated at least three times.

### *Lactate dehydrogenase (LDH) activity assay* [1]

SH-SY5Y cells were cultured with 1 mM MPP^+^ for 24 h. Then, the activity of LDH released into the culture medium was measured using an LDH assay kit (Jiancheng Institute of Bioengineering, China) according to the manufacturer’s instructions. A microplate reader (Bio-Rad, Hercules, CA, USA) was used to record the absorbance at 490 nm.

### ELISA

SH-SY5Y cells were treated with MPP^+^ for 24 h, harvested and centrifuged to detect the expression levels of TNF-α and IL-1β using an ELISA kit (BioLegend, Inc., CA, USA) according to the instructions provided by the manufacturer. A microplate reader (Bio-Rad, Hercules, CA, USA) was employed to measure the absorbance at 450 nm [1].

### Reactive oxygen species (ROS) release and superoxide dismutase (SOD) activity test

The treated cells were incubated with 10 μM 2ʹ-7ʹdichlorofluorescin diacetate (DCFH-DA) (Sigma) at 37°C for 45 min in the dark. A fluorescence microplate reader (Labsystems Oy, Helsinki, Finland) was used to quantify the fluorescence intensity, using an excitation wavelength of 485 nm and an emission wavelength of 530 nm [1].

SH-SY5Y cells were gathered and lysed using cell lysis buffer (Beyotime, Shanghai, China). According to the manufacturer’s instructions, a SOD activity assay kit (Beyotime, Shanghai, China) was used to determine the SOD activity.

### Statistical analysis

The experimental data are provided as the mean ± standard deviation of at least three independent experiments. SPSS 13.0 software was used for statistical analysis. The difference between the two groups was determined by Student’s t-test, and one-way analysis of variance followed by the Bonferroni post-hoc test was applied to analyze the difference between multiple groups. A p-value below 0.05 (P < 0.05) indicated a significant difference.

## Results

### miR-942-5p is a target of lncRNA SOX2-OT

**Figure 1.**
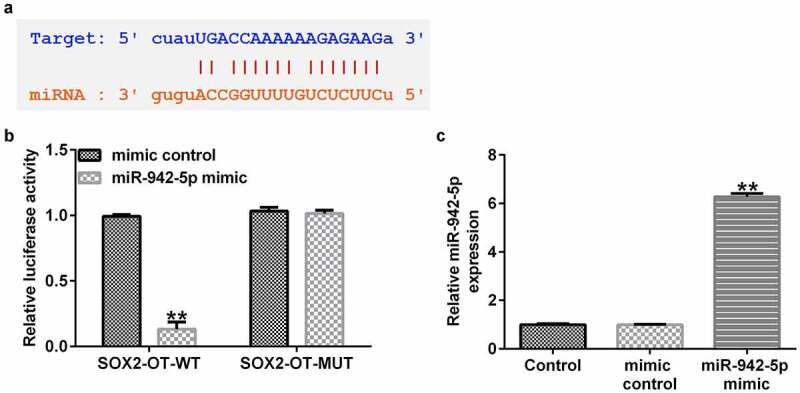
lncRNA SOX2-OT directly targeted miR-942-5p. (a) The Starbase discovered the binding site between lncRNA SOX2-OT and miR-942-5p. (b) A dual luciferase reporter assay was used to reveal the relationship between lncRNA SOX2-OT and miR-942-5p. (c) The level of miR-942-5p in 293 T cells transfected with mimic control or miR-942-5p mimic was determined using qRT-PCR

In order to explore their role in PD, the correlation between miR-942-5p and lncRNA SOX2-OT was examined. Starbase results demonstrated the binding sites between lncRNA SOX2-OT and miR-942-5p (Figure 1(a)). In addition, a luciferase reporter assay was performed in 293 T cells to verify the predicted binding sites of lncRNA SOX2-OT and miR-942-5p. The experimental results confirmed the direct targeting relationship between lncRNA SOX2-OT and miR-942-5p (Figure 1(b)). Moreover, we observed that the miR-942-5p mimic significantly enhanced miR-942-5p expression in 293 T cells (Figure 1(c)).

### MPP^+^ treatment increases lncRNA SOX2-OT and decreases miR-942-5p expression levels in SH-SY5Y cells

**Figure 2.**
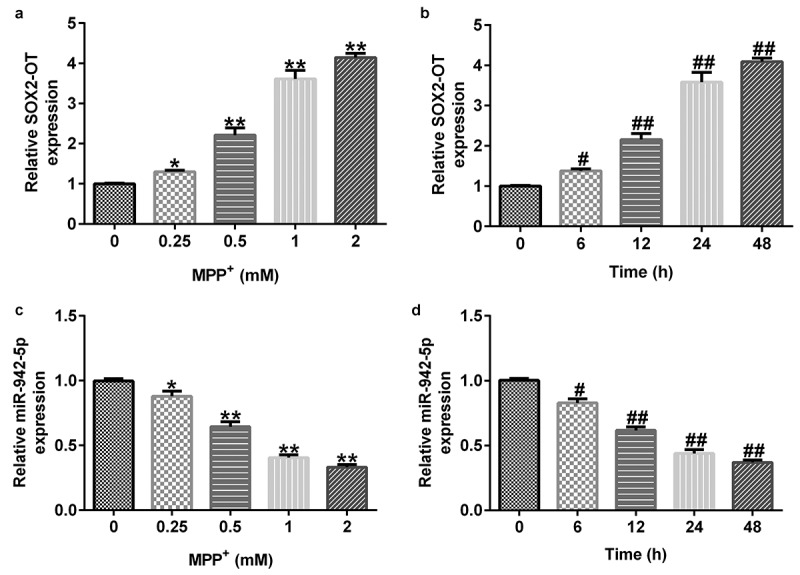
Expression of lncRNA SOX2-OT and miR-942-5p in MPP^+^-induced SH-SY5Y cells. The expression levels of SOX2-OT (a) and miR-942-5p (c) were analyzed by qRT-PCR in SH-SY5Y cells treated with different doses of MPP^+^ for 24 h. The expression levels of SOX2-OT (b) and miR-942-5p (d) determined by qRT-PCR in SH-SY5Y cells cultured with 1 mM MPP^+^ for different time periods

To further elucidate the functions of lncRNA SOX2-OT and miR-942-5p, we examined their expression levels in SH-SY5Y cells by qRT-PCR.Moreover, SH-SY5Y cells were treated with 0, 0.25, 0.5, 1, or 2 mM MPP^+^ (Sigma, St. Louis, MO, USA) for 24 h, or exposed to 1 mM MPP^+^ for 0, 6, 12, 24, or 48 h. The experimental results demonstrated that compared to the control group, MPP^+^ increased the expression level of SOX2-OT (Figure 2(a,b)) in a dose- and time-dependent manner, and reduced miR-942-5p (Figure 2(c,d)) levels in SH-SY5Y cells.

### lncRNA SOX2-OT affects miR-942-5p expression in SH-SY5Y cells

**Figure 3.**
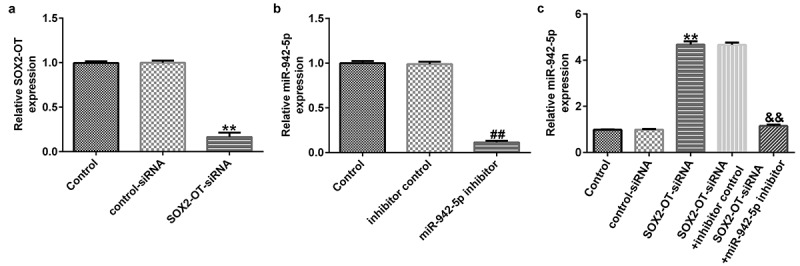
Effects of lncRNA SOX2-OT downregulation on the expression of miR-942-5p in SH-SY5Y cells. (a) SOX2-OT and (b) miR-942-5p levels in SH-SY5Y cells detected by qRT-PCR. (c) miR-942-5p levels in SH-SY5Y cells transfected with control siRNA, SOX2-OT siRNA, SOX2-OT siRNA + inhibitor control, or SOX2-OT siRNA + miR-942-5p inhibitor were detected by qRT-PCR

To explore the effects of lncRNA SOX2-OT on miR-942-5p expression, the SH-SY5Y cells were transfected with control siRNA, SOX2-OT siRNA, inhibitor, control, miR-942-5p inhibitor, SOX2-OT siRNA + inhibitor control, or SOX2-OT siRNA + miR-942-5p inhibitor for 48 h. The transfection efficiency was measured by qRT-PCR. The experimental results showed that compared with the control siRNA group, SOX2-OT-siRNA significantly reduced the expression of SOX2-OT in SH-SY5Y cells (Figure 3(a)). Compared with the inhibitor control group, the miR-942-5p inhibitor significantly decreased the expression of miR-942-5p in SH-SY5Y cells (Figure 3(b)). Compared to the control siRNA group, SOX2-OT siRNA significantly improved the expression of miR-942-5p in SH-SY5Y cells, and this effect was eliminated by the addition of miR-942-5p inhibitor (Figure 3(c)).

### lncRNA SOX2-OT influences neuronal apoptosis, inflammatory response, and the induction of oxidative stress in MPP^+^-induced SH-SY5Y cells

**Figure 4.**
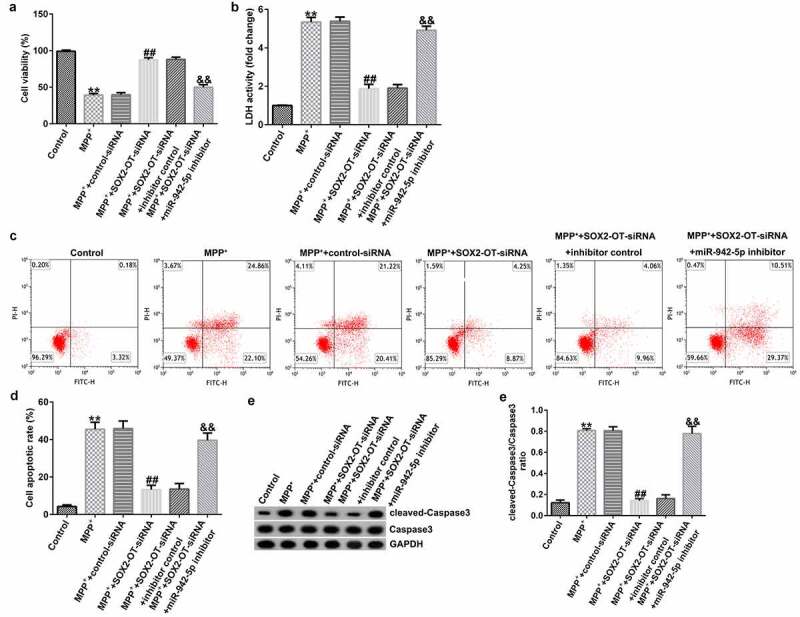
Effects of lncRNA SOX2-OT inhibition on MPP^+^-induced SH-SY5Y cell viability and apoptosis. (a) The cell viability of SH-SY5Y cells following treatments was measured by MTT assay. (b) The lactate dehydrogenase (LDH) release assay was applied to detect the LDH activity of treated SH-SY5Y cells. (c) and (d) Flow cytometry analysis was employed to detect the apoptotic rate of treated SH-SY5Y cells. (e) and (f) Western blotting was employed to examine the expression of cleaved caspase-3 protein and to calculate the ratio of cleaved caspase-3/caspase-3 of treated SH-SY5Y cells

**Figure 5.**
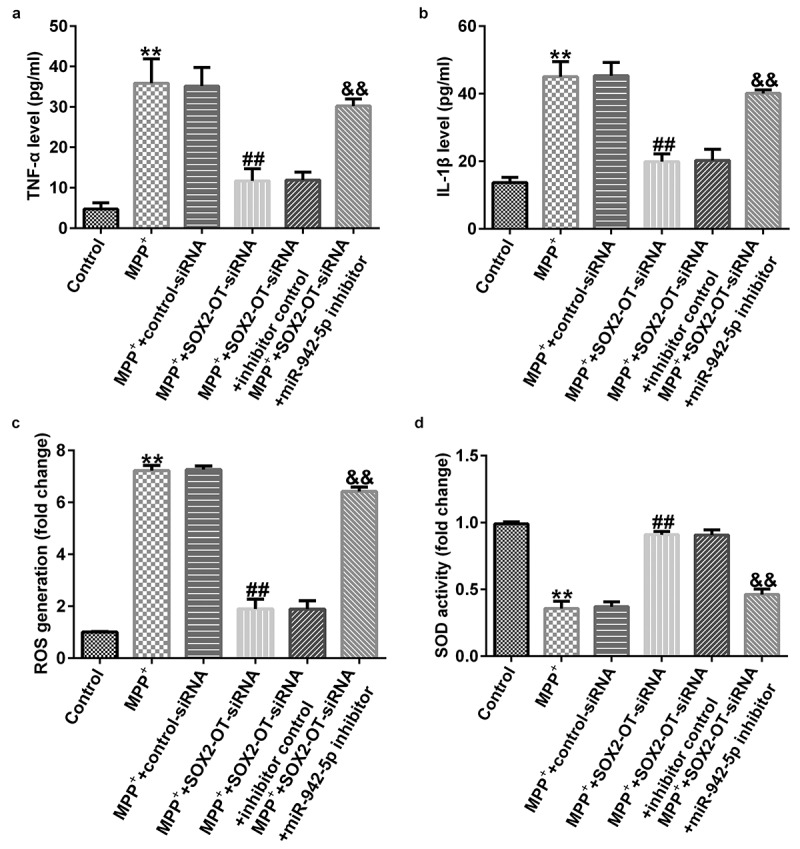
Effects of lncRNA SOX2-OT inhibition on the inflammatory response and oxidative stress in MPP^+^-induced SH-SY5Y cells. (a) and (b) TNF-α and IL-1β levels in treated SH-SY5Y cells were measured by ELISA. (c) and (d) The intracellular level of reactive oxygen species (ROS) release and superoxide dismutase (SOD) activity

To explore the mechanism of lncRNA SOX2-OT in PD, the SH-SY5Y cells were transfected with control siRNA, SOX2-OT siRNA, SOX2-OT siRNA + inhibitor control, or SOX2-OT siRNA + miR-942-5p inhibitor for 48 h, and then treated with 1 mM MPP^+^ for 24 h. These cells were divided into the following groups: control, MPP^+^, MPP^+^ + control siRNA, MPP^+^ + SOX2-OT siRNA, MPP^+^ + SOX2-OT siRNA + inhibitor control, and MPP^+^ + SOX2-OT siRNA + miR-942-5p inhibitor groups. Subsequently, MTT assay, LDH assay, and FCM was used to detect cell viability, LDH activity, and apoptosis; and western blotting was performed to estimate the protein expression of cleaved caspase-3 and to calculate the ratio of cleaved caspase-3/caspase-3. Furthermore, ELISA was used to examine the secretion of TNF-α and IL-1β, and ROS release and SOD vitality were also tested. The results revealed that, compared to the control group, MPP^+^ treatment observably decreased SH-SY5Y cell viability (Figure 4(a)), increased the activity of LDH (Figure 4(b)), induced apoptosis (Figure 4(c,d)), increased cleaved caspase-3 protein expression (Figure 4(e)), and enhanced the ratio of cleaved caspase-3/caspase-3 (Figure 4(f)). Contrastingly, compared with the MPP^+^ + control siRNA treatment group, SOX2-OT siRNA increased the viability of SH-SY5Y cells, decreased the activity of LDH, reduced cell apoptosis, decreased cleaved caspase-3 protein expression, and reduced the ratio of cleaved caspase-3/caspase-3, and these effects were significantly eliminated by miR-942-5p inhibitor. Moreover, MPP^+^ treatment significantly increased the levels of TNF-α (Figure 5(a)), IL-1β (Figure 5(b)), and ROS (Figure 5(c)) in SH-SY5Y cells, and decreased SOD activity (Figure 5(d)). Compared to the MMP^+^ + control siRNA group, SOX2-OT siRNA significantly reduced the levels of TNF-α (Figure 5(a)), IL-1β (Figure 5(b)), and ROS (Figure 5(c)) in SH-SY5Y cells, and increased SOD activity (Figure 5(d)). These effects were significantly eliminated by miR-942-5p inhibitor.

### NAIF1 is a target of miR-942-5p

**Figure 6.**
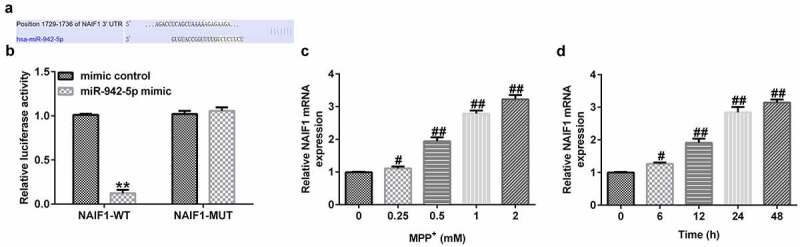
miR-942-5p directly targeted NAIF1 and the expression of NAIF1 in MPP^+^-induced SH-SY5Y cells. (a) The TargetScan discovered the binding site between miR-942-5p and NAIF1. (b) Dual luciferase reporter assay was used to reveal the relationship between miR-942-5p and NAIF1. (c) The expression levels of NAIF1 were analyzed by qRT-PCR in SH-SY5Y cells treated with different doses of MPP^+^ for 24 h. D. The expression levels of NAIF1 were analyzed by qRT-PCR in SH-SY5Y cells treated with 1 mM MPP^+^ for different times

In order to study the molecular mechanism of miR-942-5p and NAIF1 in PD, the relationship between miR-942-5p and NAIF1 was examined. TargetScan results indicated the binding sites between miR-942-5p and NAIF1 (Figure 6(a)). Moreover, a luciferase reporter assay was performed in 293 T cells in order to ascertain the predicted binding sites of NAIF1 and miR-942-5p. The experimental results confirmed the direct targeting relationship between miR-942-5p and NAIF1 (Figure 6(b)).

In addition, the expression level of NAIF1 was detected by qRT-PCR in SH-SY5Y cells treated with MPP^+^. Moreover, SH-SY5Y cells were treated with 0, 0.25, 0.5, 1, or 2 mM MPP^+^ (Sigma, St. Louis, MO, USA) for 24 h, or exposed to 1 mM MPP^+^ for 0, 6, 12, 24, or 48 h. The experimental results revealed that, compared to the control group, MPP^+^ increased the mRNA expression level of NAIF1 (Figure 6(c,d)) in a dose- and time-dependent manner in SH-SY5Y cells.

### miR-942-5p negatively regulates NAIF1 expression in SH-SY5Y cells

**Figure 7.**
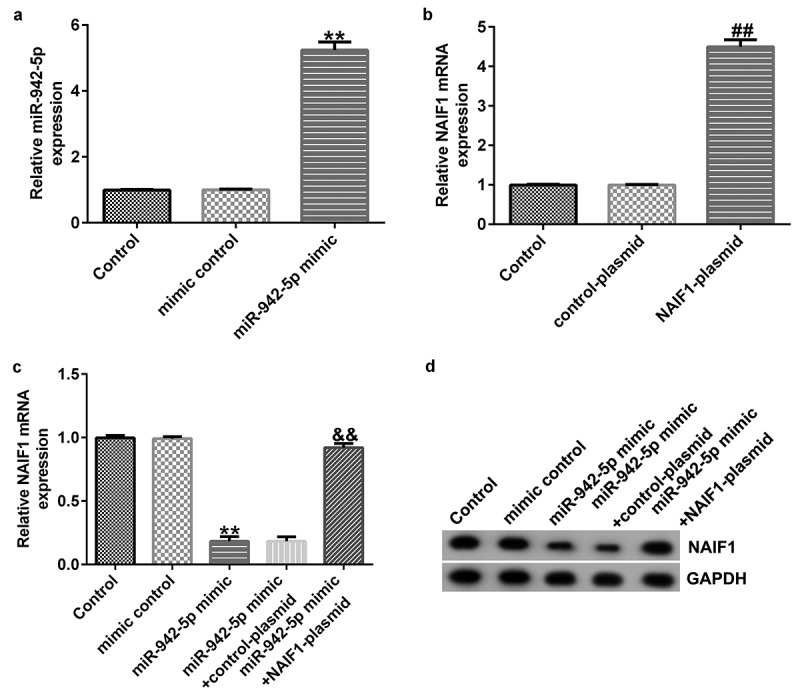
Effects of miR-942-5p upregulation on the expression of NAIF1 in SH-SY5Y cells. (a) The levels of miR-942-5p in SH-SY5Y cells were examined by qRT-PCR. (b) The mRNA levels of NAIF1 in treated SH-SY5Y cells were detected by qRT-PCR assay. (c) The mRNA and protein levels of NAIF1 in SH-SY5Y cells transfected with mimic control, miR-942-5p mimic, miR-942-5p mimic + control plasmid, or miR-942-5p mimic + NAIF1 plasmid were detected by qRT-PCR and western blotting assays, respectively

To explore the contribution of miR-942-5p and NAIF1 to the development of PD, the SH-SY5Y cells were transfected with mimic control, miR-942-5p mimic, control plasmid, NAIF1 plasmid, miR-942-5p mimic + control plasmid, or miR-942-5p mimic + NAIF1 plasmid for 48 h. The transfection efficiency was determined by qRT-PCR and western blotting. The experimental results revealed that, compared to the mimic control group, the miR-942-5p mimic observably increased the expression of miR-942-5p in SH-SY5Y cells (Figure 7(a)). Compared to the control plasmid group, the NAIF1 plasmid observably increased the expression of NAIF1 mRNA in SH-SY5Y cells (Figure 7(b)). Compared with the mimic control group, the miR-942-5p mimic significantly reduced the NAIF1 mRNA and protein levels in SH-SY5Y cells, and these reductions were significantly eliminated by the NAIF1 plasmid (Figure 7(c,d)).

### miR-942-5p influences neuronal apoptosis, inflammatory response, and the induction of oxidative stress in MPP^+^-induced SH-SY5Y cells

**Figure 8.**
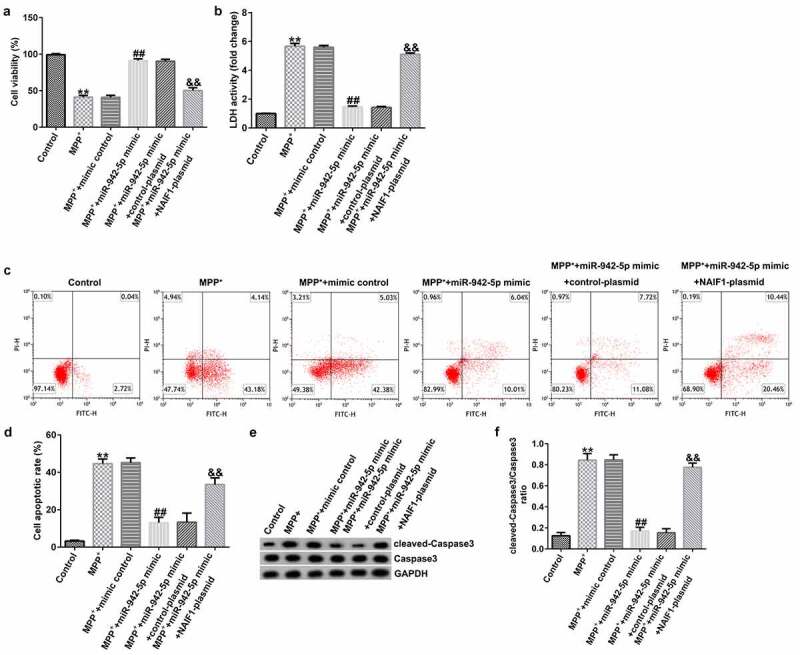
Effects of miR-942-5p on MPP^+^-induced SH-SY5Y cell viability and apoptosis. (a) The cell viability in SH-SY5Y cells following treatments was measured by MTT assay. (b) The lactate dehydrogenase (LDH) release assay was applied to detect the LDH activity of treated SH-SY5Y cells. (c) and (d) Flow cytometry was employed to detect the apoptotic rate of treated SH-SY5Y cells. (e) and (f) Western blotting was employed to examine the expression of cleaved caspase-3 protein and to calculate the ratio of cleaved caspase-3/caspase-3 in treated SH-SY5Y cells

To explore the mechanism of miR-942-5p affecting PD, the SH-SY5Y cells were transfected with mimic control, miR-942-5p mimic, miR-942-5p mimic + control plasmid, or miR-942-5p mimic + NAIF1 plasmid for 48 h, and then treated with 1 mM MPP^+^ for 24 h. These cells were divided into the following groups: Control, MPP^+^, MPP^+^ + mimic control, MPP^+^ + miR-942-5p mimic, MPP^+^ + miR-942-5p mimic + control plasmid, and MPP^+^ + miR-942-5p mimic + NAIF1 plasmid groups. The results showed that, compared with the MPP^+^ + mimic control treatment group, the miR-942-5p mimic significantly increased SH-SY5Y cell viability (Figure 8(a)), reduced the activity of LDH (Figure 8(b)), reduced cell apoptosis (Figure 8(c,d)), reduced cleaved caspase-3 protein expression (Figure 8(e)), and reduced the ratio of cleaved caspase-3/caspase-3 (Figure 8(f)), and all of these effects were significantly eliminated by the NAIF1 plasmid.

**Figure 9.**
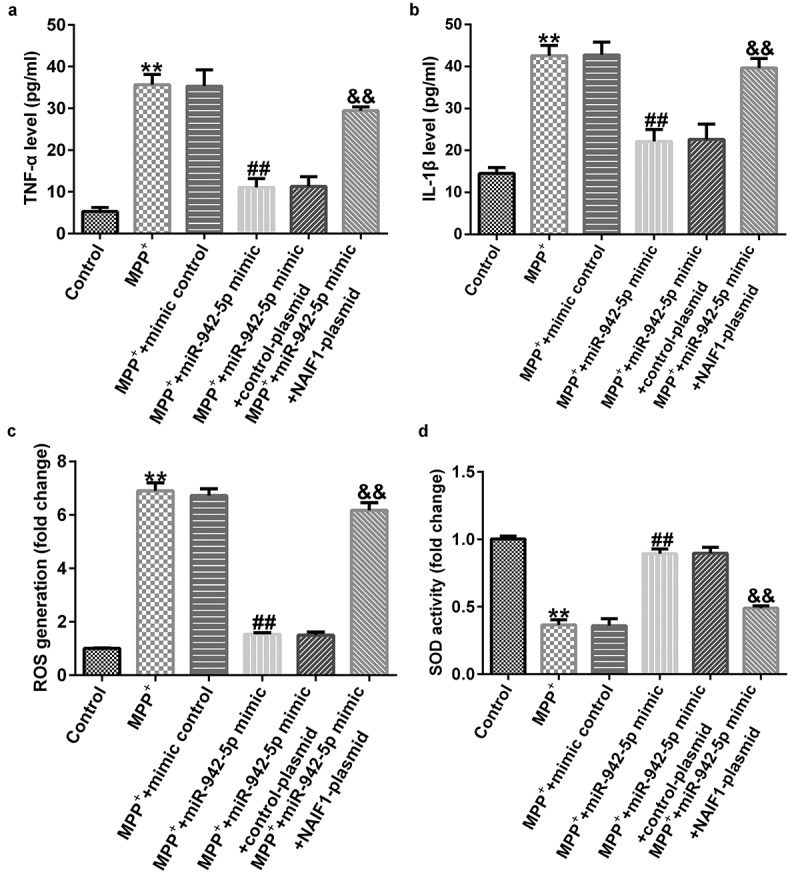
Effects of miR-942-5p on the inflammatory response and oxidative stress in MPP^+^-induced SH-SY5Y cells. (a) and (b) The levels of TNF-α and IL-1β in treated SH-SY5Y cells were measured by ELISA. (c) and (d) The intracellular level of reactive oxygen species (ROS) release and superoxide dismutase (SOD) activity

Compared to the MMP^+^ + mimic control group, the miR-942-5p mimic significantly reduced the levels of TNF-α (Figure 9(a)), IL-1β (Figure 9(b)), and ROS (Figure 9(c)) in SH-SY5Y cells, and increased SOD activity (Figure 9(d)). These effects were significantly eliminated by the NAIF1 plasmid.

## Discussion

lncRNAs have appeared as a novel regulator of neurogenesis [27]. SOX2OT is a lncRNA that harbors one of the primary regulators of pluripotency [28]. Recently, studies have proven that lncRNA is expressed at significantly high levels in PD patients [29–32]. Notably, inflammation, oxidative stress, and neuronal apoptosis are also related to the pathogenesis of PD. A group of studies have proven that miRNAs can participate in the development of various diseases by regulating the expression levels of key factors for cell growth and apoptosis [33–35]. Studies have also reported the abnormal expression and important role of miRNA in PD [36,37]. Moreover, a targeting relationship between NAIF1 and certain miRNAs has also been reported [38]. This evidence indicates that lncRNA, in association with miRNAs and NAIF1, may participate in the development of PD, but the specific mechanism remains unclear.

In this study, the prediction analysis demonstrated that lncRNA SOX2-OT directly targeted miR-942-5p and NAIF1. This study revealed that the expression level of lncRNA SOX2-OT and NAIF1 increased significantly, while that of miR-942-5p decreased following an increase in the concentration levels of MPP^+^ and in the treatment period, which is similar to the results of previous studies [1,^22^]. In addition, the effects of lncRNA SOX2-OT, miR-942-5p, and NAIF1 were studied using the *in vitro* PD cell model. MPP^+^ is a commonly used substance for establishing PD in the *in vitro* models [39]. The results demonstrated that the downregulation of the expression level of lncRNA SOX2-OT significantly increased SH-SY5Y cell viability, reduced cell apoptosis, and decreased LDH viability in MPP^+^-treated SH-SY5Y cells, and these effects were significantly eliminated by miR-942-5p inhibitor. Apoptosis or necrosis can cause cell membrane rupture, resulting in the release of LDH. The activity of the released LDH can represent the amount of cell necrosis and is widely used in cytotoxicity testing [40]. Furthermore, preceding studies have shown that proinflammatory factors are related to the pathogenesis of PD [41]. In the current study, ELISA was employed to examine IL-1β and TNF-α and other pro-inflammatory factors, and western blotting was employed to estimate the expression levels of cleaved caspase-3 protein. The results showed that the downregulated expression of lncRNA SOX2-OT leads to a decrease in the expression levels of TNF-α, IL-1β, and cleaved caspase-3 protein in SH-SY5Y cells treated with MPP^+^ and reduces the cleaved caspase-3/caspase-3 ratio. In addition, we further noticed that the downregulated expression of lncRNA SOX2-OT significantly reduced the ROS levels and enhanced the SOD activity in MPP^+^-treated SH-SY5Y cells in the *in vitro* PD cell model. These results showed that lncRNA SOX2-OT inhibition suppresses MPP^+^-induced oxidative stress; however, the miR-942-5p inhibitor significantly reversed all the above effects in SH-SY5Y cells treated with MPP^+^. Further, we observed that the miR-942-5p mimic significantly improved SH-SY5Y cell viability and reduced cell apoptosis, cleaved caspase-3 protein expression, ratio of cleaved caspase-3/caspase-3, LDH activity, and the levels of TNF-α, IL-1β, and ROS in SH-SY5Y cells, and increased the activity of SOD, but these effects were significantly eliminated by the NAIF1 plasmid. These results indicate that lncRNA SOX2-OT modulates the inflammatory response and induction of oxidative stress and neuronal apoptosis by targeting miR-942-5p and NAIF1 in an *in vitro* PD cell model.

## Conclusion

In summary, this study shows that lncRNA SOX2-OT regulates inflammation, oxidative stress, and neuronal apoptosis by directly regulating the miR-942-5p/NAIF1 signal axis, thereby participating in the occurrence and development of PD. The results of this study provide a novel potential target for PD diagnosis and therapy.

## Data Availability

The datasets used and/or analyzed during the current study are available from the corresponding author upon reasonable request.

## Highlights

- MPP^+^ treatment increased lncRNA SOX2-OT and decreased miR-942-5p expression levels in SH-SY5Y cells.
- lncRNA SOX2-OT down-regulation relieved MPP^+^-induced SH-SY5Y cells injury via up-regulating miR-942-5p.
- miR-942-5p relieved MPP^+^-induced SH-SY5Y cells injury via down-regulating NAIF1 expression.
